# Overlay of conventional angiographic and *en-face *OCT images enhances their interpretation

**DOI:** 10.1186/1471-2415-5-12

**Published:** 2005-06-13

**Authors:** Mirjam EJ van Velthoven, Koos de Vos, Frank D Verbraak, Chris W Pool, Marc D de Smet

**Affiliations:** 1 Department of Ophthalmology, Academic Medical Centre, University of Amsterdam, Meibergdreef 9, 1105 AZ Amsterdam, the Netherlands; 2 Netherlands Institute for Brain Research, Meibergdreef 33, 1105 AZ, Amsterdam, the Netherlands

## Abstract

**Background:**

Combining characteristic morphological and functional information in one image increases pathophysiologic understanding as well as diagnostic accuracy in most clinical settings. *En-face *optical coherence tomography (OCT) provides a high resolution, transversal OCT image of the macular area combined with a confocal image of the same area (OCT C-scans). Creating an overlay image of a conventional angiographic image onto an OCT image, using the confocal part to facilitate transformation, combines structural and functional information of the retinal area of interest. This paper describes the construction of such overlay images and their aid in improving the interpretation of OCT C-scans.

**Methods:**

In various patients, e*n-face *OCT C-scans (made with a prototype OCT-Ophthalmoscope (OTI, Canada) in use at the Department of Ophthalmology (Academic Medical Centre, Amsterdam, The Netherlands)) and conventional fluorescein angiography (FA) were performed. ImagePro, with a custom made plug-in, was used to make an overlay-image. The confocal part of the OCT C-scan was used to spatially transform the FA image onto the OCT C-scan, using the vascular arcades as a reference. To facilitate visualization the transformed angiographic image and the OCT C-scan were combined in an RGB image.

**Results:**

The confocal part of the OCT C-scan could easily be fused with angiographic images. Overlay showed a direct correspondence between retinal thickening and FA leakage in Birdshot retinochoroiditis, localized the subretinal neovascular membrane and correlated anatomic and vascular leakage features in myopia, and showed the extent of retinal and pigment epithelial detachment in retinal angiomatous proliferation as FA leakage was subject to blocked fluorescence. The overlay mode provided additional insight not readily available in either mode alone.

**Conclusion:**

Combining conventional angiographic images and *en-face *OCT C-scans assists in the interpretation of both imaging modalities. By combining the physiopathological information in the angiograms with the structural information in the OCT scan, zones of leakage can be correlated to structural changes in the retina or pigment epithelium. This strategy could be used in the evaluation and monitoring of patients with complex central macular pathology.

## Background

The combined use of morphological and functional information has been shown to improve diagnostic accuracy in many clinical disciplines [1-3]. In the management of patients with malignancies, coronary heart disease and diseases of the brain, fusion of different image modalities, such as CT, MRI, PET and various other nuclear diagnostic tests, is widely used [3-7]. Retinal diseases are also very complex, and often more than one diagnostic or imaging technique is used to assist in making a diagnosis. Cunha-Vaz and co-workers [8,9] have shown the value of multimodal mapping systems for the macula, incorporating retinal imaging techniques such as confocal scanning laser ophthalmoscopy (SLO), the retinal leakage analyzer, the retinal thickness analyzer, automated perimetry and SLO angiography. However, inherent difficulties exist in the ability to correlate these various modalities, as these techniques lack common, precise reference points.

Recently, a new modality in ocular imaging was introduced combining high depth resolution OCT with high transversal resolution confocal ophthalmoscopy [10-13]. The prototype OCT-Ophthalmoscope simultaneously produces *en-face *OCT scans and pixel-to-pixel corresponding confocal images, so called OCT C-scans. The OCT C-scans are oriented in a transversal plane, perpendicular to the optical axis of the eye. A stack of such OCT C-scans allows for a reconstruction of the retina in three dimensions. Although this transversal plane is the more familiar plane when viewing the retina, compared to the conventional, longitudinal OCT images [14-16], single *en-face *OCT scans may look confusing to the uninitiated, due to their high depth resolution (about 10 micron) and their transversal orientation through the retina [12,17].

The confocal channel creates a fundoscopic image with a high transversal resolution but a low depth resolution (~300 μm). There is seemingly little change in the confocal image when scanning through the retina along the Z-axis (depth). Therefore the confocal part of the OCT C-scan provides a high quality fundus image throughout the whole OCT stack in depth. As both the confocal and OCT image are acquired simultaneously with the same light source and at the same scanning rate, there is pixel-to-pixel correspondence between the OCT and the confocal image of the OCT C-scan [11]. The confocal image is used for general orientation and localization, whereas the OCT image shows detailed morphology, and subsequent pathological changes within the retina [10].

The high quality confocal image makes it a reliable reference image to overlay the OCT C-scans onto images generated with other diagnostic techniques acquired in the transversal plane. For example, with appropriate software to implement an affine transformation, conventional angiographic images can be spatially transformed and then superimposed over the confocal image. Given the pixel-to-pixel correspondence between the confocal and OCT channel, the converted angiographic image can be directly superimposed over the OCT image as well. The fusion of the more familiar angiographic information with the highly detailed morphological information from the OCT C-scan could improve our knowledge of retinal diseases as visualized by *en-face *OCT. In this paper, we describe this novel procedure, and assess its ability to enhance our understanding of both fluorescein angiography and OCT C-scans through the combination of structural and functional information.

## Methods

At the Department of Ophthalmology of the Academic Medical Centre (University of Amsterdam, Amsterdam, The Netherlands) a prototype OCT-Ophthalmoscope (Ophthalmic Technologies Inc., Toronto, Canada) was used to evaluate patients with various retinal pathologies. Patients undergoing routine fluorescein angiography (FA), using Imagenet (Topcon, USA), were scanned with the OCT-Ophthalmoscope in the area of interest on the same day. To demonstrate the possibilities of this new technique, we selected some illustrative, though not necessarily common, pathological cases.

The configuration of this OCT prototype has been described previously [10,13]. In short, the system uses a super luminescent diode, with a central wavelength of 820 nm (20 nm bandwidth). The light beam is split, directing one part to the patient's eye (sample arm) and the other part to a reference arm (mirror). The returning light beams from both the patient's eye and the reference arm are collected through an interferometer to produce the OCT signal [11]. A fraction of the light returning from the patients' eye is also directed towards another detector to produce a confocal signal [11]. The OCT-Ophthalmoscope produces a transversal OCT C-scan (size: 1042 × 512 pixels), currently at 2 frames/second, in the X-Y-plane at a fixed Z-coordinate, in which the OCT and confocal images are in pixel-to-pixel correspondence with each other [17].

**Figure 1:**
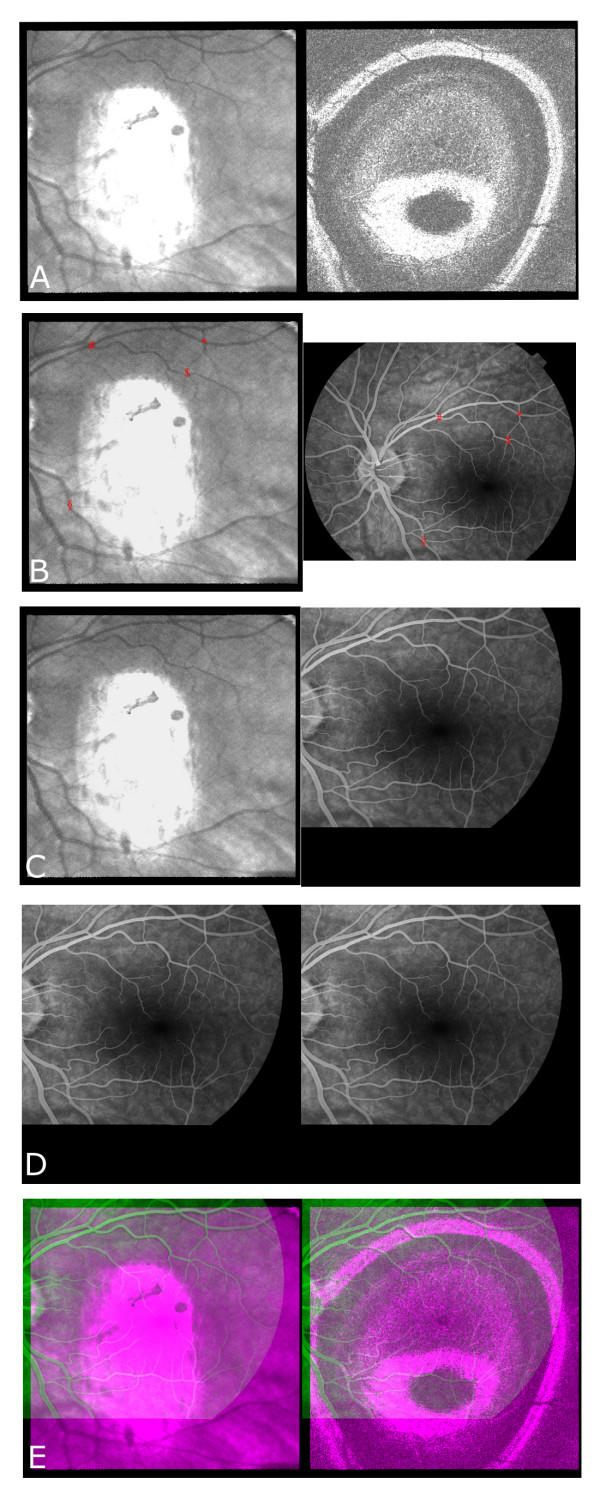
**Procedure used in overlay technique using images of a normal fundus**. A) original OCT C-scan of normal fundus taken at midretina (large white area in confocal part (left) = corneal reflection); B) Confocal part of OCT C-scan (left) and midfase FA image (right), red markers represent reference points used for spatial transformation; C) Confocal part of OCT C-scan (left) and transformed FA image (right); D) duplicated converted FA image; E) overlay image: green = FA and red/blue = OCT C-scan, showing on the left side the achieved registration based on the confocal part and the FA image, and on the right side the fusion with the OCT part.

ImagePro is an image-processing program working under Windows^©^. By using a custom made plug-in for this program, it is possible to spatially transform images, and to superimpose them over reference images. For the purpose of this study, the confocal part (size: 512 × 512 pixels) of the OCT C-scan was used as a reference image. Since every transformation inherently leads to loss of anatomical resolution in the transformed image, we chose to leave the OCT C-scan intact. The OCT C-scan covers a smaller area than the FA. Detailed information in the OCT scan would be lost due to a necessary volume transformation if the OCT C-scan would be transformed onto the FA. One or two OCT C-scans were chosen from the stack, depending on the depth at which the pathologies were best visible. The appropriate angiographic Imagenet frame was transformed onto, and superimposed over the confocal image. Alignment was then achieved by using evident landmarks as reference points. Inherent to the scanning procedure of the OCT-Ophthalmoscope, using a curved fan ray equivalent to the curvature of the eye, there is some distortion at the borders of both parts of OCT C-scan relative to the centre of the images. We found that it was best to choose reference points centrally within the vascular arcades. Four to six pairs of reference points were manually selected. This was done by zooming in on the area in which a landmark was localised. Landmarks were chosen at vascular crossings or vessel bifurcations that were clearly visible in both image modalities. These reference points were used to compute a linear transformation by the least-square method, compensating for translation, rotation, scaling, and shearing (full affine). The spatially transformed angiographic image was doubled and then superimposed over both the confocal image and the OCT scan. To further facilitate visualization, the images were combined into the different layers of an RGB image: the green channel of the RGB image was used for the spatially transformed angiographic image, and the other channels were used for both parts of the OCT C-scan. Images presented in this paper show on the left-hand side the confocal image, and on the right-hand side the OCT image with or without angiographic overlay. Figure 1 shows the above described procedure for making an overlay image with the help of OCT C-scan and FA images of a normal fundus. The whole procedure (including acquisition and selection of the appropriate image frames) took no more than 15 minutes per patient.

## Results

### Example A: Birdshot retinochoroiditis (figure 2)

**Figure 2:**
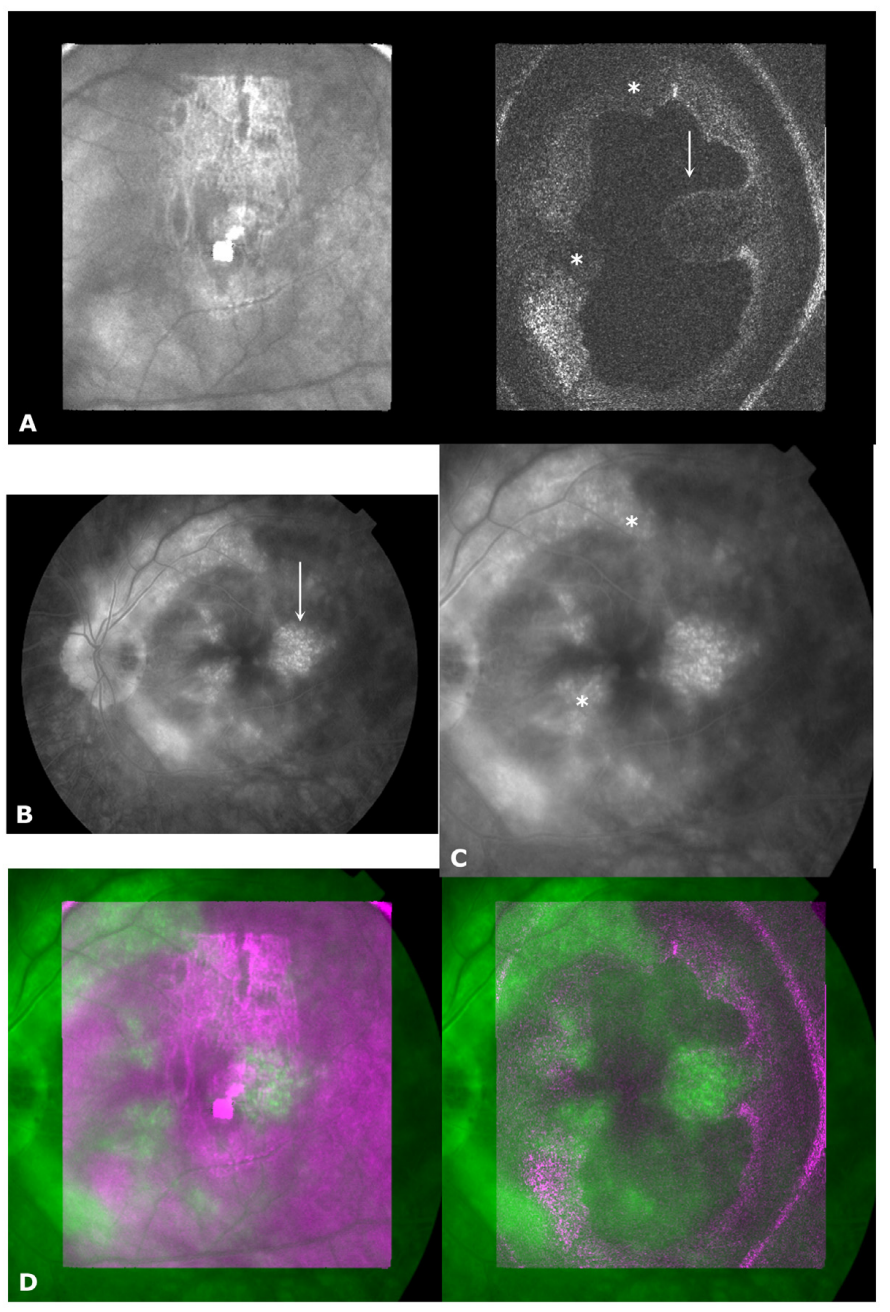
**Birdshot retinochoroiditis**. A) OCT C-scan taken pre-foveal. Right side (OCT part): arrow = temporal retinal thickening, * = irregularly shaped retinal contour. Left side (confocal part): large patchy white area = corneal reflection, bright central white spot = reflection from system lenses; B) FA late phase image, arrow: temporal leakage; C) transformed FA image, * = diffuse leakage nasally and along arcades; D) overlay image, red/blue = OCT C-scan, green = FA.

The OCT C-scan shows marked retinal swelling temporal to the fovea (figure 2a), with small cystoid changes. On the late FA image (figure 2b) leakage is seen along the vascular arcades and in the fovea. The spatially transformed FA image (figure 2c) is superimposed over the OCT C-scan (figure 2d). This overlay image shows that the temporal area of retinal thickening on the OCT exactly matches the temporal, parafoveal area of leakage on the angiographic image. Reviewing the overlay image more closely, smaller areas of extensive leakage at the nasal side of the fovea and along the vascular arcades on the angiographic image (figure 2c, asterisks) correspond to the somewhat irregularly shaped retinal contour on the OCT C-scan (figure 2a, asterisks). Generalized retinal thickening along the arcades corresponds to extensive intraretinal vascular leakage on the angiogram. There is correspondence between a physiologic parameter (leakage) and morphological changes (retinal thickening and cystic changes).

### Example B: high myopia with secondary neovascularization (figure 3)

**Figure 3:**
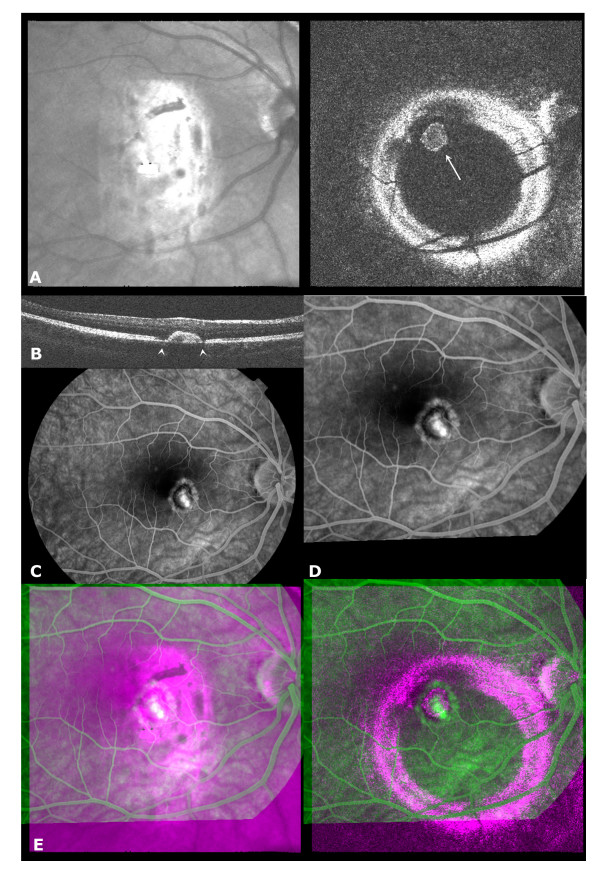
**High myopia with secondary neovascularization**. A) OCT C-scan taken at level of RPE, arrow (right) = neovascular membrane surrounded by a hyporeflective halo (left: patchy central white area in confocal part = corneal reflection); b) *en-face *OCT B-scan through neovascular membrane, arrowheads indicate disruption of the double highly reflective layer at the level of RPE caused by the changed orientation of the photoreceptor layer; C) FA mid-phase image showing inside-out a central hyperfluorescence, a hypofluorescent halo (hyperpigmentation) and a halo of hyperfluorescence (window defect) in the area of the neovascularization; D) transformed FA image; E) overlay image showing that the central hyperfluorescence on FA is enclosed by the RPE detachment on OCT, and that the hyperfluorescent halo corresponds to the hyporeflective halo in the OCT.

Figure 3a shows an OCT C-scan at the level of the retinal pigment epithelium (RPE) in a highly myopic patient with a solitary neovascular membrane, located just outside the foveolar area. An irregularly shaped, hyperreflective circular line is seen, corresponding to a pigment epithelial detachment (PED). The content of this circular area is dense, suggesting that it contains a highly reflective tissue such as a neovascular membrane rather than serous fluid as might be seen in central serous retinopathy [18]. In addition, on the OCT C-scan, the distinct sharp border generated by the PED is surrounded by a dark halo. On the OCT B-scan, the inner band of the double highly reflective layer at the level of the RPE is interrupted just before the edges of the PED (figure 3b), corresponding to the dark halo on the OCT C-scan. The dark halo on the OCT C-scan and the interruption of this inner highly reflective layer in the OCT B-scan is most likely caused by the changed orientation of the photoreceptor outer segments. This changes the reflectivity properties of the photoreceptor layer and therefore the reflectivity profile in the OCT image. The FA image (figure 3c) shows very prominent leakage in the centre of the lesion, surrounded by a first halo of hypofluorescence, and a second halo of hyperfluorescence. Good spatial correspondence is seen in the overlay (figure 3e). The bright spot of prominent leakage on the FA is located well within the confinement of the PED as seen in the OCT C-scan. The hypofluorescent halo on the FA corresponds to the hyperreflective circle on OCT delineating the RPE surrounding the neovascular membrane. The outer hyperfluorescent zone on the FA corresponds to the hyporeflective halo on the OCT. This hyperfluorescent zone on the FA shows the characteristics of a window defect. The corresponding hyporeflective halo in the OCT C-scan surrounding the membrane can be a result of this loss of pigmentation, but is more likely caused by changed reflectivity of the photoreceptor layer due to its changed orientation, as supported by figure 3b. Both OCT B- and C-scan also clearly show that the membrane is located below the RPE, which makes it ineligible for current surgical techniques.

### Example C: Retinal Angiomatous Proliferation (RAP, figure 4)

**Figure 4:**
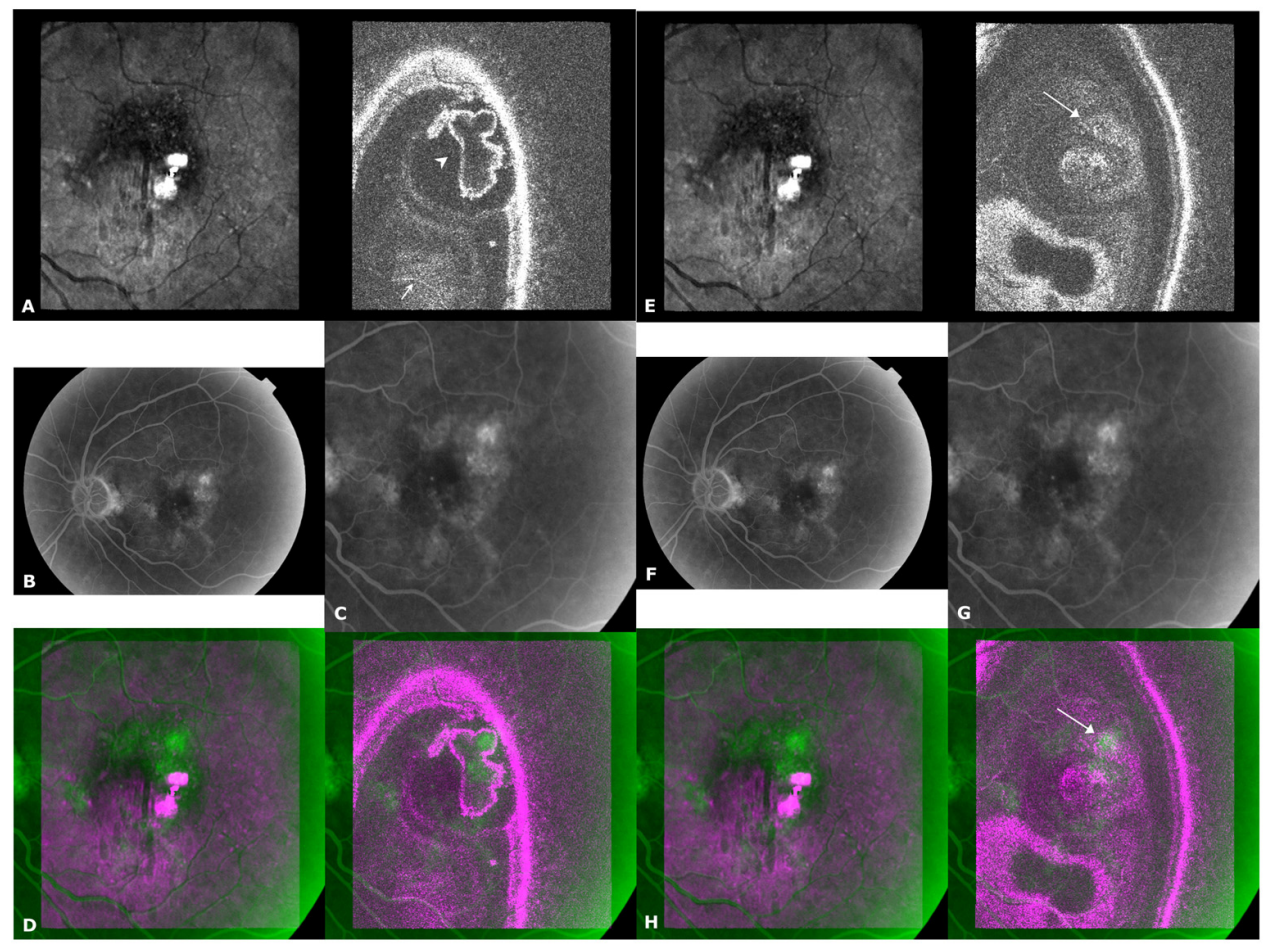
**Retinal Angiomatous Proliferation (RAP)**. A) C-scan taken at midretina, small arrow = retinal vessel, arrowhead = large irregular pigment epithelial detachment, arrowhead placed within serous retinal detachment; B) FA image; C) transformed FA image; D) Overlay image showing leakage on FA to be located at the centre of the serous/epithelial detachments on OCT, though its vertical location within the 3-dimensional RAP cannot be specified; E-H) second overlay image created from a C-scan taken midretina, in which the bright spot on FA seems to correlate with the high reflective area in the OCT C-scan (arrows). This is most likely the crest of the irregularly shaped PED or of the retinal component of the angiomatous lesion.

This patient has a stage II RAP based on the FA image (figure 4b) [19]. Reviewing the FA image, a large area of hyperfluorescence is seen surrounding a bright, fluorescent central spot, presumably the RAP. The first OCT C-scan (figure 4a, right) is taken at the level of the retinal vessels. It shows a large serous neurosensory detachment and a large, very irregularly shaped PED. The overlay shows a discrepancy between the hyperfluorescence in the FA and the structural information in the OCT image (figure 4d). The hyperfluorescence seems mostly confined to a part of the PED seen on the OCT. The bright spot within the area of diffuse hyperfluorescence can be correlated with a highly reflective area in a second OCT C-scan taken above the large PED (figure 4e–h). This appeared to correspond to the top of the PED, but more likely relates to a highly reflective proliferation located between the neurosensory retina and the RPE (not shown).

## Discussion

The OCT-Ophthalmoscope provides clinicians with a novel imaging modality of the retina. The transversally oriented OCT C-scans allow software-assisted overlay with more traditional imaging techniques, such as FA or indocyanine green (ICG) angiography, that are also displayed in a transversal plane. The confocal channel in the OCT-Ophthalmoscope provides a fundoscopic image with a high transversal resolution which has a pixel-to-pixel correspondence with the OCT channel that possesses a very high depth resolution [10]. Therefore, the confocal image can easily be used as a reference image. Images produced by other diagnostic techniques can be mathematically transformed to fit snugly over the confocal image of the OCT C-scan, and thereby be directly superimposed over the OCT image.

Our initial goal in making overlay images, was to enhance the interpretation of individual OCT C-scans. These are initially difficult to interpret. The high transversal resolution makes recognition of retinal landmarks difficult in the OCT C-scan images. Classically, pathologic processes are studied in longitudinal sections, so that their transversal appearance is rarely appreciated. By using the overlay technique, it is possible to correlate pathologic features imaged with familiar transversal imaging techniques, such FA, with the morphological information provided on OCT C-scans. We also found that the overlay technique provided additional insight not readily available with either modality alone.

In our example of Birdshot retinochoroiditis (figure 2) the OCT C-scan showed an irregularly shaped retinal contour in the parafoveolar area, and along the arcades. On the FA image diffuse parafoveolar oedema was present as well as diffuse leakage along the major retinal vessels. Leakage correlated well in the overlay to the irregularly shaped retinal contour on the OCT. Thus, the patchy retinal thickening seen on OCT was caused by diffuse oedema, which if needed could be followed prospectively, without the need of the more invasive angiographic technique. In figure 3, OCT and FA concurred in the location of the neovascular membrane. Further, the OCT also showed that the neovascular membrane was situated below the RPE, making this membrane ineligible for current surgical procedures [20], while the retina above was intact and not involved in the process. Combining findings on the longitudinal and transversal OCT allowed one to better comprehend the FA manifestations observed at the edge of the neovascular lesion. Hypofluorescence at the edge of the PED is due to superposition of pigmented RPE cells, while the more peripheral hyperfluorescence is caused by a window defect. The OCT and FA images in the RAP example (figure 4) revealed the limitations of FA in defining the extent of involvement, in particular of the RPE [21,22]. FA leakage was present in only a portion of the PED, probably due to the presence of blocked fluorescence, although the cause of this blocked fluorescence could not be ascertained with the OCT.

Conventional OCT is a valuable tool in making a diagnosis and in monitoring the progress of macular diseases. The commercially available *Stratus*OCT scans in a longitudinal orientation, providing high resolution, morphologic, cross-sectional OCT scans of the retina, but it does not provide a precise localization of the scanned lines within the macular region, because there is always a time delay between acquisition of the line and storage of the line with its associated fundus image. A retinal thickness map is based on the interpolation of the thickness measurements from six radial OCT scan lines. While these maps are useful to monitor patients, they cannot be combined with other transversal diagnostic techniques because both the map and the scan lines which it is made of, lack good reference points. For this and other reasons, conventional OCT has not yet been incorporated in multimodal mapping systems such as the one developed by Cunha-Vaz and co-workers [8].

Rosen and co-workers [23] were able to produce simultaneous ICG angiography and *en-face *OCT in an adjusted, experimental OCT-Ophthalmoscope. Here pixel-to-pixel correspondence was seen in both channels, which ultimately may be a better set-up than the technique proposed here. However, this combination will need further optimization before it becomes clinically applicable. At this moment, our examples suggest that multimodal mapping is possible between the OCT-Ophthalmoscope and existing imaging modalities, without various imaging modalities being incorporated into the system. Combining several imaging techniques provides complementary information which helps in interpreting and understanding the findings of each modality. Additional modalities which could possibly be fused with transversal OCT include microperimetry and (multifocal) electroretinography. These would provide functional correlations to structural alterations in the retina.

## Conclusion

Combining conventional angiographic images and *en-face *OCT C-scans assists in the interpretation of both imaging modalities. By combining the physiopathological information in the angiograms with the structural information in the OCT scan, zones of leakage can be correlated to structural changes in the retina or RPE. Fusion of different imaging modalities can contribute to our understanding of complex retinal or choroidal diseases and it can optimize our patient management.

## Competing interests

The author(s) declare that they have no competing interests.

## Authors' contributions

MvV carried out the acquisition of the OCT images, made the software-assisted overlay images and was responsible for writing this paper. KdV participated extensively in acquiring the overlay images and contributed to writing this paper, especially the methodology section. FV participated in the design of this study, contributed to the interpretation of the acquired overlay images and to writing the paper. CP participated in the design of this study and coordinated the acquisition of the overlay images. MdS participated in the design and coordination of this study, contributed in interpreting the images and in writing the paper. All authors read and approved the final manuscript.

## Pre-publication history

The pre-publication history for this paper can be accessed here:


